# Mosquito Bed Net Use and Burkitt Lymphoma Incidence in Sub-Saharan Africa: A Systematic Review and Meta-Analysis

**DOI:** 10.1001/jamanetworkopen.2024.7351

**Published:** 2024-04-18

**Authors:** Nora Schmit, Jeevan Kaur, Elom K. Aglago

**Affiliations:** 1 MRC Centre for Global Infectious Disease Analysis, Imperial College London, London, United Kingdom; 2 Faculty of Medicine, Imperial College London, London, United Kingdom; 3 Department of Epidemiology and Biostatistics, Imperial College London, School of Public Health, London, United Kingdom; 4 Faculty of Science and Technology, University of Kara, Kara, Togo

## Abstract

**Importance:**

Burkitt lymphoma (BL) is one of the most common childhood cancers in sub-Saharan Africa and is etiologically linked to malaria. However, evidence for an effect of malaria interventions on BL is limited.

**Objective:**

To investigate the potential population-level association between large-scale rollout of insecticide-treated bed nets (ITNs) in sub-Saharan Africa in the 2000s and BL incidence.

**Data Sources:**

In this systematic review and meta-analysis, a search was conducted in the Embase, Global Health, and Medline databases and in cancer registry publications between January 1, 1990, and February 27, 2023.

**Study Selection:**

All epidemiologic studies on BL incidence rates in children and adolescents aged 0 to 15 years in sub-Saharan African countries where malaria is endemic were identified by 2 reviewers blinded to each other’s decision.

**Data Extraction and Synthesis:**

The systematic review was conducted according to the Preferred Reporting Items for Systematic Reviews and Meta-analyses reporting guideline. Data were extracted independently by 2 reviewers, and quality was scored based on 3 predefined criteria: data collection, case ascertainment, and calculation of person-time at risk.

**Main Outcomes and Measures:**

Incidence rates of BL during childhood and mean ITN use in the population. Data were analyzed using a random-effects negative binomial regression model.

**Results:**

Of 2333 studies meeting selection criteria, 23 comprising 66 data points on BL incidence were included based on 5226 BL cases from locations with large-scale ITN use in 17 countries. Rates of BL were 44% (95% CI, 12%-64%) lower in the period after ITN introduction compared with before. The adjusted pooled incidence rates of BL were 1.36 (95% CI, 0.88-2.10) and 0.76 (95% CI, 0.50-1.16) per 100 000 person-years before and after introduction of ITNs, respectively. After adjusting for potential confounders, a 1–percentage point increase in mean ITN use in the population in the 10 years before BL data collection was associated with a 2% (95% CI, 1%-4%) reduction in BL incidence.

**Conclusions and Relevance:**

In this systematic review and meta-analysis, large-scale rollout of ITNs in the 2000s was associated with a reduction in BL burden among children in sub-Saharan Africa. Although published data may not be representative of all incidence rates across sub-Saharan Africa, this study highlights a potential additional benefit of malaria control programs.

## Key Points

**Question:**

Is there a population-level association between large-scale rollout of insecticide-treated bed nets (ITNs) in the 2000s and Burkitt lymphoma (BL) incidence rates in children in sub-Saharan Africa?

**Findings:**

In this systematic review and meta-analysis of 5226 BL cases from 23 studies, a 1–percentage point increase in 10-year mean ITN use in the population was associated with a 2% reduction in BL incidence after adjusting for potential population-level confounders.

**Meaning:**

These results suggest that large-scale introduction of ITNs in the 2000s was associated with a reduction in childhood BL burden in sub-Saharan Africa.

## Teaser

This systematic review and meta-analysis investigates whether large-scale use of insecticide-treated bed nets is associated with reduced incidence of Burkitt lymphoma among children in sub-Saharan Africa.

## Introduction

Burkitt lymphoma (BL) is an aggressive type of non-Hodgkin lymphoma and among the most common childhood cancers in sub-Saharan Africa.^1^ Infection with Epstein-Barr virus is an established risk factor for BL, and there is epidemiologic and biologic evidence for an etiologic relationship between *Plasmodium falciparum* malaria and BL.^1,2^ Malaria still poses an important public health problem, with over 200 million clinical cases worldwide annually.^3^ Malaria parasite prevalence and the cumulative number of infections correlate with BL incidence at the population level,^4,5^ and individual-level associations between malaria infection or antimalarial antibody titers and BL risk have been reported.^6^

This combined evidence raises the possibility that interventions against malaria might have downstream beneficial effects on reducing the burden of BL. In an uncontrolled malaria suppression trial from 1977 to 1982 in Tanzania, a decline in BL incidence rates was observed during a mass chloroquine distribution campaign, although the decline started before the trial years and rates increased again afterward.^7^ Two hospital-based case-control studies suggested that using mosquito nets or living in a household with insecticide use could potentially decrease the risk of developing BL in African children,^8,9^ while a recent population-based case-control study reported an increased risk of BL in children exposed to mass malaria suppression (use of a mosquito net the previous night or living in households with indoor residual insecticide spraying in the past year).^10^ These mixed results may indicate a difficulty in accounting for residual confounding from malaria burden, as interventions are preferentially targeted at high-transmission areas with the highest BL incidence, and for the temporal relationship between intervention exposure and BL development.^2,10^

The 2000s marked a period of unprecedented donor funding and the first time that malaria interventions were widely rolled out in sub-Saharan Africa.^3^ Mass campaigns focused on distributing insecticide-treated bed nets (ITNs) and artemisinin-based combination therapies.^3^ These control efforts were associated with reducing *P falciparum* infection prevalence in sub-Saharan Africa by an estimated 50% between 2000 and 2015, with most of this decline attributable to ITNs.^11^ In this study, we aimed to investigate the association between this large-scale rollout of ITNs in the early 2000s and a potential decrease in BL incidence in sub-Saharan Africa using an ecological study design. First, we conducted a systematic review to assemble all publicly available data on BL incidence rates in sub-Saharan African children since 1990. We calculated pooled estimates of BL incidence across sub-Saharan African countries where malaria is endemic before and after large-scale ITN introduction and compared time trends in incidence in individual locations. Second, we used model estimates of subnational ITN use^12^ to investigate the association between mean ITN use in the population and BL incidence in children.

## Methods

### Systematic Review

This systematic review and meta-analysis was conducted using cancer registry publications and in literature databases. We searched websites associated with the International Agency for Research on Cancer (IARC), which collates data from population-based cancer registries globally.^13,14,15^ Using the Ovid platform, we also searched the Embase, Global Health, and Medline databases up to February 27, 2023, for all studies on BL incidence rates in sub-Saharan Africa published since January 1, 1990, without language restrictions. Search terms are shown in eTable 1 in Supplement 1. This study was registered in PROSPERO and was reported according to the Preferred Reporting Items for Systematic Reviews and Meta-analyses (PRISMA) reporting guideline.^16^ Full methodologic details can be found in the eMethods in Supplement 1.

Epidemiologic studies reporting the incidence rate of BL in children and adolescents aged 0 to 15 years in sub-Saharan African countries where malaria is endemic were included. Incidence rates had to apply to the general population. The outcome of interest was incident cancer cases diagnosed as BL in a defined population and period with a midpoint after 1990, and eligible studies also had to report the person-time at risk or the crude incidence rate.

Articles were deduplicated and screened in Covidence. Screening was performed independently by 2 reviewers (N.S., J.K.) blinded to each other’s decision, and disagreements were resolved through discussion. Data were extracted independently by the 2 reviewers into a prevalidated template in Excel, version 2401 (Microsoft Corporation), with any differences resolved in a final assessment. Harmonized extracted data included location, time, study design, data collection and ascertainment methods, age range, number of cases, person-years at risk, and incidence rate. Studies identified from the published literature were cross-checked against the dataset extracted from cancer registry publications for duplicates and overlap in data from the same registry. Their quality was scored based on 3 predefined criteria (data collection, case ascertainment, and calculation of person-time at risk).

### Statistical Analysis

Crude BL incidence rates in children and adolescents aged 0 to 15 years were converted to units of cases per 100 000 person-years. Due to the rarity of the cancer, BL incidence is usually reported for aggregate periods. In the analysis, rates were assumed to apply to the calendar midpoint of the data collection period.

#### Time Trends in BL Incidence

To assess time trends and generate pooled incidence rates for sub-Saharan Africa in the period before and after large-scale ITN introduction, we estimated the year of ITN introduction for each location with BL data. The Malaria Atlas Project generates comparable model estimates of yearly ITN use since 2000 at a fine spatial resolution.^12^ Based on information about the covered population in each study, we assigned each BL data point to its corresponding first administrative-level unit. The urbanicity status of the population covered by the study was used to split ITN use estimates into urban and rural areas within each unit using a threshold density of 1500 people per square kilometer. We calculated the mean ITN use weighted by the population at risk in each unit over time.

Use of ITNs has generally increased since 2000, but temporal patterns vary between locations (eFigure 2 in Supplement 1). To capture the beginning of large-scale, population-wide ITN campaigns, we defined the year of ITN introduction as the first year with at least 5% use and an increasing trend. Several southern African countries had nationwide BL data available but did not introduce ITNs at a large scale (mean ITN use <1%) (eFigure 2 in Supplement 1).^17^ These countries used other malaria interventions (eg, artemisinin-based combination therapies and indoor residual spraying)^17^; therefore, we considered their data separately.

Incidence rates of BL in each group were analyzed using a negative binomial regression model with person-time at risk as an offset and including location-level random intercepts. The pooled BL incidence rate with 95% CI was estimated for each group and for individual countries. Additionally, for the subset of locations with at least 1 BL data point before and after ITN introduction, we calculated the rate ratio for BL incidence after ITN introduction compared with before to assess trends for each location individually.

#### Population-Level Association Between ITN Use and BL Incidence

To investigate the association between mean ITN use in the population and BL incidence in children, we considered different time lags for population-level exposure to ITNs. The latency period between malaria exposure and BL onset is not fully understood; most children in areas where malaria is highly endemic have their first exposure to both Epstein-Barr virus and malaria in the first few years of life,^18^ while BL is most common in children aged 5 to 10 years.^1^ This observation led to the hypothesis that repeated malaria infections during childhood would increase the risk of BL.^5^ Given this and the variable patterns in ITN scale-up, we hypothesized that ITN use in a particular year would be a poor indicator of BL incidence and instead used the mean population ITN use in the 10 years before BL data collection as the measure of exposure to capture the lifetime exposure to ITNs in the majority of BL cases. In sensitivity analyses, we also tested models with mean population ITN use in the 5 or 15 years before BL data collection as well as concurrent ITN use. We assumed no exposure to ITN use in the population before 2000.^3^

We identified several a priori potential confounders for the population-level association between ITN use and BL incidence: baseline malaria prevalence in 2000 specific to the first administrative-level unit (before implementation of interventions) and concurrent prevalence of HIV infection in adults, the country-specific Human Development Index (HDI), and the urbanicity status of the population covered by the cancer registry or study (Table 1^3,12,19,20,21,22,23,24,25,26,27,28^ and eFigure 1 in Supplement 1). We calculated crude and adjusted rate ratios (RRs) for these factors using a negative binomial model with person-time at risk as the offset and including geographic location as a random intercept to account for potential correlation in data points collected in the same area at different time points (eTable 6 in Supplement 1). We identified influential outliers in the regression model based on a Cook distance greater than 4 divided by the total number of studies. In sensitivity analyses, we removed each influential outlier in turn from the model, restricted the dataset to locations with BL data before and after ITN introduction, and evaluated the association between BL incidence and the change in malaria prevalence since the baseline year (2000) as an alternative exposure.

**Table 1.** Overview of Model Covariates and Data Sources^a^

Covariate (source)	Before ITN introduction		After ITN introduction	
	Data points, No. (%) (N = 66)	Median (IQR)	Data points, No. (%) (N = 66)	Median (IQR)
Mean population ITN use over 10 y, % (Malaria Atlas Project,^12^ foresite R package^19^)^b^	35 (53)	0.0 (0.0-0.8)	31 (47)	17.6 (11.0-29.0)
Baseline malaria parasite prevalence in 2000, % (Malaria Atlas Project,^20^ foresite R package^19^)^c^	35 (53)	26.3 (21.9-49.8)	31 (47)	36.1 (19.4-51.4)
Concurrent prevalence of HIV infection in people aged 15-49 y, % (Dwyer-Lindgren et al,^21^ 2019)^d^	35 (53)	7.8 (2.5-15.2)	31 (47)	5.3 (2.9-8.4)
Concurrent country HDI level (United Nations Human Development Reports^22^)^e^				
<0.45	22 (33)	NA	9 (14)	NA
≥0.45	13 (20)	NA	22 (33)	NA
Population urbanicity status (Burkitt lymphoma data sources)^f^				
Urban	16 (24)	NA	18 (27)	NA
Both	14 (21)	NA	12 (18)	NA
Rural	5 (8)	NA	1 (2)	NA

Abbreviations: HDI, Human Development Index; ITN, insecticide-treated bed net; NA, not applicable.

^a^ Summary statistics cover the 66 locations in sub-Saharan Africa where malaria is endemic that had data on Burkitt lymphoma incidence and large-scale ITN use.

^b^ Mean yearly location-specific ITN use in the population in the 10 years before (and inclusive of) the midpoint year of the Burkitt lymphoma data point; ITN use refers to the proportion of people who sleep under a net.

^c^ Location-specific *Plasmodium falciparum* parasite prevalence in children aged 2 to 10 years in 2000. The year 2000 is commonly considered as representing baseline endemicity levels before large-scale intervention use and marking the beginning of a historically unprecedented decline in malaria burden.^23^ Malaria prevalence correlates with Burkitt lymphoma incidence and is a key consideration in targeting malaria control programs.

^d^ Location-specific prevalence of HIV infection in individuals aged 15 to 49 years in the midpoint year of the Burkitt lymphoma data point as a proxy for prevalence of HIV infection in children. HIV infection is a risk factor for Burkitt lymphoma outside sub-Saharan Africa, including in patients treated with antiretroviral therapy.^24^ HIV treatment program scale-up also occurred in the 2000s and is supported by the same international funder as malaria.^3,25^

^e^ Country-specific HDI level in the midpoint year of the Burkitt lymphoma data point. The HDI is a summary measure of human development, including life expectancy, education, and income, and was categorized into 2 levels at the median value in the dataset due to an observed nonmonotonic relationship between HDI and Burkitt lymphoma incidence. The HDI correlates with cancer patterns^26^ and could be associated with access to malaria interventions.

^f^ Urbanicity status of the population covered by the cancer registry or study. Most population-based cancer registries are located in cities and cover urban populations,^27^ while malaria is more common in rural areas.^28^

All analyses were conducted in R, version 4.2.2 (R Project for Statistical Computing) using the glmmTMB (version 1.1.7) and ggeffects (version 1.2.3) packages.^29,30,31^ *P* values were obtained from a 2-tailed Wald test, and significance was set at *P* < .05. Malaria Atlas Project estimates of ITN use were extracted from the foresite package of R (version 0.1.0).^19^

## Results

### Study Characteristics

**Figure 1.**
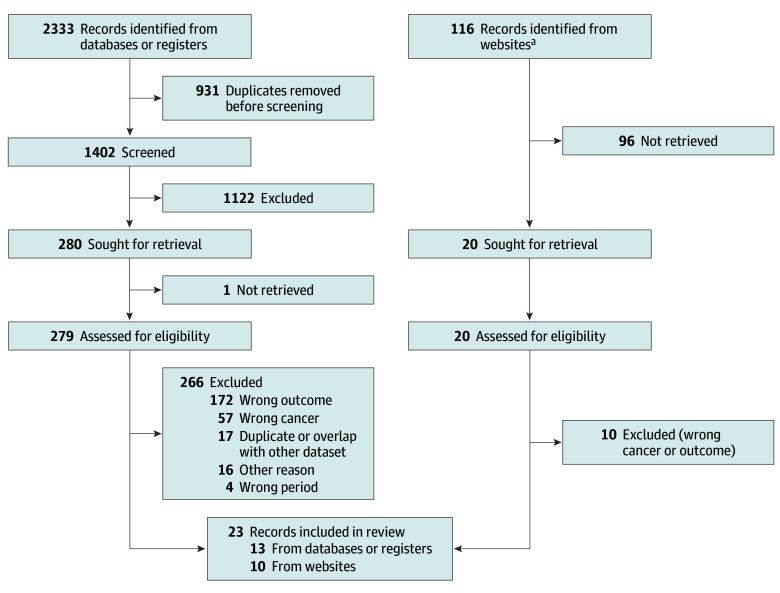
Flow Diagram of the Systematic Review on Burkitt Lymphoma Incidence Rates ^a^Websites associated with the International Agency for Research on Cancer (IARC) included IARC Publications, the Global Cancer Observatory, and the African Cancer Registry Network.

The literature search returned 2333 articles and 116 records identified on IARC-associated websites (Figure 1). After screening and deduplication of the 2 sets of data sources, a total of 13 publications from literature databases and 10 IARC reports were included.^1,5,27,32,33,34,35,36,37,38,39,40,41,42,43,44,45,46,47,48,49,50,51^ One article reported BL incidence in individuals aged 0 to 19 years while otherwise meeting inclusion criteria, but unpublished data in the group aged 0 to 15 years could not be obtained on request.^4^

A total of 76 location- and time-specific BL incidence rates based on a total of 5700 cases were extracted from the 23 publications^1,5,27,32,33,34,35,36,37,38,39,40,41,42,43,44,45,46,47,48,49,50,51^ (eTable 2 in Supplement 1). Common reasons for low-quality scores included passive case findings and inconsistent or poorly defined confirmations of diagnosis (eTable 3 in Supplement 1). Included data covered the period from 1990 to 2017, with slightly more data points available after 2005 and 61 of 76 (80%) obtained from population-based cancer registries. The BL incidence rates came from 32 locations in 21 countries (eFigure 3 in Supplement 1). Analyses relating to ITN use were restricted to 66 data points based on 5226 BL cases from locations with large-scale ITN use and covering 17 countries.

### Time Trends in BL Incidence

The median year of ITN introduction in the included locations was 2005 (range, 2004-2011). The BL incidence was estimated to be 44% (95% CI, 12%-64%) lower in the period after large-scale ITN introduction compared with before. Across countries, adjusted pooled incidence rates were 1.36 (95% CI, 0.88-2.10) and 0.76 (95% CI, 0.50-1.16) per 100 000 person-years for the periods before and after ITN introduction, respectively (Table 2).^1,5,27,32,33,34,35,36,37,38,39,40,41,42,43,44,45,46,47,48,49,50,51^ The adjusted pooled rate was lower in countries without large-scale ITN use (0.18 [95% CI, 0.07-0.46] per 100 000 person-years). However, there was also large variation in the estimated incidence rates between countries, ranging from 0.09 (95% CI, 0.02-0.37) to 5.98 (95% CI, 3.82-9.37) per 100 000 person-years before ITN introduction and from 0.07 (95% CI, 0.01-0.35) to 8.52 (95% CI, 2.75-26.33) per 100 000 person-years after ITN introduction (Table 2).

**Table 2.** Country-Level Pooled Estimates of Burkitt Lymphoma Incidence

Country	Location	Period	Data points, No.^a^	Cases, No.	Person-time at risk	Adjusted incidence rate, per 100 000 person-years (95% CI)^b^
**Before introduction of ITNs (1990-2008)**							
Cameroon^35,43,47^	Northwest Province, Yaounde	2003-2006, 2007-2009, and 2004-2006	3	239	6 275 949	4.36 (2.57-7.39)
Congo^44^	Brazzaville	1996-1999	1	11	1 017 088	1.08 (0.38-3.11)
Cote d’Ivoire^44^	Abidjan	1995-1997	1	49	3 788 100	1.29 (0.52-3.24)
Guinea^1,44^	Conakry	1993-1995, 1996-1999, and 2001-2010	3	52	9 029 258	0.75 (0.41-1.34)
Kenya^1,38,41,44,47^	Eldoret, nationwide, and Nyanza Province	1998-2006, 1988-1992, 1993-1997, and 1999-2004	4	1263	134 918 246	1.20 (0.77-1.89)
Malawi^33,44,46,50^	Blantyre	1991-1995, 1996-1998, 1999-2001, and 2003-2007	4	403	5 472 399	5.98 (3.82-9.37)
Mali^44^	Bamako	1988-1997	1	3	3 388 015	0.09 (0.02-0.37)
Mozambique^36^	Maputo	1991-2008	1	133	7 130 923	1.87 (0.76-4.55)
Niger^1,44,45^	Niamey	1993-1999, 2001-2005	2	29	3 366 189	0.87 (0.42-1.78)
Nigeria^44,45^	Ibadan	1993-1999, 2006-2009	2	68	3 534 946	1.96 (1.01-3.80)
Tanzania^32^	Mwanza, Mara	2000-2004	1	540	10 114 350	5.34 (2.22-12.86)
The Gambia^1,44,45,48^	National	1988-1996, 1997-1998, and 2002-2006	3	45	7 965 430	0.63 (0.34-1.15)
Uganda^40,42,48,49,50^	Northern Uganda, Kyadondo County	1997-2001, 2002-2006, 1989-1991, 1993-1997, 1998-2002, and 2003-2007	6	836	38 319 546	2.57 (1.78-3.72)
Zambia^34^	Nationwide	1990-1992	1	11	9 166 667	0.12 (0.04-0.34)
Zimbabwe^44,49,50^	Harare	1990-1997, 1998-2006	2	15	8 308 590	0.18 (0.08-0.40)
Total	NA	1990-2008	35	3697	251 795 696	1.36 (0.88-2.10)
**After introduction of ITNs (2007-2017)**							
Benin^27^	Cotonou	2014-2016	1	4	735 900	0.54 (0.12-2.39)
Congo^27,45^	Brazzaville	2009-2016	1	6	4 606 275	0.13 (0.03-0.51)
Cote d’Ivoire^27,45^	Abidjan	2012-2013, 2014-2015	2	118	6 008 792	1.98 (0.88-4.43)
Ethiopia^1,27^	Addis Ababa	2011-2016	1	3	4 207 728	0.07 (0.01-0.35)
Kenya^1,5,27,45^	Eldoret, Nairobi, and Northwestern Kenya	2007-2011, 2007-2011, 2012-2014, and 2012-2016	4	224	27 487 675	0.60 (0.33-1.08)
Malawi^1,50^	Blantyre	2008-2010	1	102	1 197 708	8.52 (2.75-26.33)
Mali^27,45,47^	Bamako	2005-2009, 2010-2014, and 2015-2017	3	121	10 353 780	1.25 (0.64-2.43)
Mozambique^27,37,39,45^	Beira, Maputo, and northern region	2009-2013, 2014-2017, 2014-2017, and 2015	4	35	7 730 296	0.54 (0.27-1.07)
Niger^45^	Niamey	2006-2009	1	9	1 717 564	0.52 (0.14-1.90)
Nigeria^27,45^	Abuja, Calabar, and Ekiti	2013-2016, 2009-2013, 2016-2017, and 2013-2017	4	13	4 426 697	0.43 (0.19-0.97)
Tanzania^5,27,32^	Mwanza, Mara	2005-2009, 2012-2015, and 2016-2017	3	504	37 787 627	1.63 (0.84-3.16)
The Gambia^45^	National	2007-2011	1	6	3 723 130	0.16 (0.04-0.63)
Uganda^5,27,51^	Kyadondo County, northern region	2008-2013, 2010-2016	2	359	18 867 124	1.84 (0.83-4.08)
Zambia^27^	Lusaka	2011-2015	1	13	4 246 505	0.31 (0.09-1.06)
Zimbabwe^1,27,50^	Bulawayo, Harare	2013-2015, 2007-2015	2	12	5 181 909	0.30 (0.10-0.87)
Total	NA	2007-2017	31	1529	138 278 710	0.76 (0.50-1.16)
**Countries without large-scale ITN use (1990-2017)**							
Botswana^1,47^	National	1999-2008	1	4	6 334 397	0.06 (0.02-0.19)
Eswatini^27,44^	National	1989-1999, 2016-2017	2	12	2 484 569	0.45 (0.23-0.90)
Namibia^27,44,45^	National	1995-1998, 2009, and 2013-2015	3	12	5 945 760	0.20 (0.10-0.38)
South Africa^1,27,44,45,47^	National	2003-2007, 2008-2012, 2013-2016, 1989-1992, 1998-2006, 2007, and 2008-2014	4	446	303 370 823	0.14 (0.11-0.19)
Total	NA	1991-2017	10	474	318 135 549	0.18 (0.07-0.46)

Abbreviations: ITN, insecticide-treated net; NA, not applicable.

^a^ The number of data points can refer to different periods or different studies in the same location.

^b^ Calculated using a negative binomial model and adjusted for clustering in data from the same geographic location.

**Figure 2.**
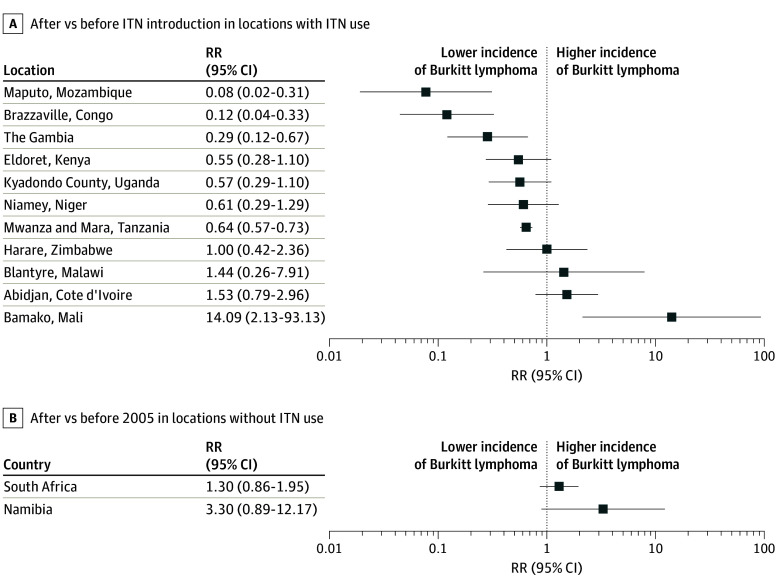
Rate Ratios (RRs) for Burkitt Lymphoma Incidence After vs Before Insecticide-Treated Bed Net (ITN) Introduction in Different Cancer Registries or Study Locations Rate ratios are shown on a logarithmic scale for all locations with at least 1 data point in both periods. B, Namibia and South Africa did not have large-scale ITN use but were separated into 2 periods: before and after 2005 (the median year of ITN introduction).

In locations with at least 1 data point before and after ITN introduction, RRs were highly variable, and time trends were not always consistent across locations (Figure 2 and eFigure 4 in Supplement 1). The incidence rate appeared to be lower in the period after ITN introduction in 7 of 12 locations, but the 95% CIs overlapped in 3 of these. The largest reductions (71%-92%) were seen in Maputo, Mozambique; Brazzaville, Congo; and The Gambia. These locations also had among the highest mean ITN use since 2000 (over the 75th percentile at 18%, 15%, and 19%, respectively). In Bamako in Mali, the incidence rate from 2005 to 2017 was significantly higher than that from 1988 to 1997. In the 2 registries without large-scale ITN use (Namibia and South Africa), rates were slightly higher after 2005 than before, but there was no significant difference between the 2 periods.

### Population-Level Association Between ITN Use and BL Incidence

Table 3 shows the crude and adjusted associations of BL incidence rates with mean population ITN use over 10 years and potential confounders. Univariate and multivariate analyses showed comparable results. There was a population-level association between ITN use and BL incidence rates after adjusting for baseline malaria prevalence, prevalence of HIV infection in adults, country HDI level, and population urbanicity (Table 3). A 1–percentage point increase in mean ITN use was associated with a 2% (95% CI, 1%-4%) reduction in BL incidence, as shown in eFigure 5 in Supplement 1. Baseline malaria prevalence was positively associated with BL incidence after adjusting for all other covariates, but there was no association with the other variables (Table 3).

**Table 3.** Negative Binomial Regression Models for the Association Between Burkitt Lymphoma Incidence and ITN Use^a^

Covariate	Crude rate ratio (95% CI)	*P* value	Adjusted rate ratio (95% CI)^b^	*P* value
Mean population ITN use over 10 y^c^	0.98 (0.96-0.99)	.003	0.98 (0.96-0.99)	.005
Baseline malaria parasite prevalence in 2000	1.02 (1.00-1.04)	.03	1.02 (1.01-1.04)	.01
Prevalence of HIV infection in people aged 15-49 y	1.02 (0.96-1.08)	.54	1.01 (0.96-1.07)	.61
Country HDI level				
<0.45	1 [Reference]	NA	1 [Reference]	NA
≥0.45	0.75 (0.43-1.29)	.29	0.95 (0.56-1.62)	.85
Population urbanicity status				
Urban	1 [Reference]	NA	1 [Reference]	NA
Both	1.70 (0.77-3.74)	.19	1.25 (0.60-2.59)	.55
Rural	1.92 (0.48-7.72)	.36	2.11 (0.64-7.00)	.22

Abbreviations: HDI, Human Development Index; ITN, insecticide-treated bed net; NA, not applicable.

^a^ The analysis was conducted on 66 incidence estimates based on 5226 cases and a total population at risk of 390 074 406.

^b^ Adjusted for all other covariates in the table.

^c^ Represents the mean ITN use in the population at the first administrative level unit in the 10 years before the Burkitt lymphoma data collection period.

Comparing the main result with different exposure periods for ITN use, the association was similar for mean ITN use in the 5 and 15 years before the BL data point. In line with our hypothesis, concurrent ITN use in the population was not associated with BL incidence (eTable 4 in Supplement 1). Additional sensitivity analyses did not affect conclusions about the association between ITN use and BL incidence (eTables 5 and 6 in Supplement 1).

## Discussion

In this study, we investigated changes in BL incidence in sub-Saharan African children in relation to large-scale introduction of ITNs in the early 2000s. In a systematic review, we identified 66 data points on BL incidence from locations with large-scale ITN use comprising 5226 BL cases across 17 countries between 1990 and 2017. We found that BL rates were 44% (95% CI, 12%-64%) lower in the period after ITN introduction compared with before. Pooled incidence rates were estimated at 1.36 (95% CI, 0.88-2.10) and 0.76 (95% CI, 0.50-1.16) per 100 000 person-years before and after ITN introduction, respectively. There was an association between increasing ITN use in the population and decreases in BL incidence among children after adjusting for potential population-level confounders.

Previous studies have also noted a decreasing temporal trend in incidence, both for BL^5,32,52,53^ and childhood non-Hodgkin lymphoma overall.^53^ Given existing evidence for an etiologic role of malaria infection in BL development,^1,2^ a population-level association between ITN use and BL is plausible. Insecticide-treated nets offer both individual and community-wide protection from mosquito bites, thereby reducing malaria prevalence.^54^ As previously suggested for malaria infection, our results also point toward past cumulative rather than concurrent exposure to ITNs as a factor associated with lower BL incidence.^5,8^

However, alternative factors could explain changes in BL incidence over time. The variable time trends in individual registries suggest other influences on observed BL incidence that limit comparability of data from different periods, locations, and sources. The coverage, completeness, and diagnostic methods of a registry can vary substantially over time. In Mali, BL incidence rates appeared to be higher after ITN introduction, but the rate before ITN introduction was based on only 3 cases from a 10-year period. Underdiagnosis of childhood cancers was suspected at the time,^44^ with subsequent improvement in case-finding procedures reflected in total cancer numbers.^27^ Population-based cancer registries in Africa have also reported increases in incidence of other cancer types in adults over time.^55,56,57^ Even though access to diagnosis might generally be expected to improve and lead to higher rates, other factors, such as intermittent political instability or funding disruption, could also contribute to variation in rates.^27,56,58^ Despite improvements, cancer surveillance in sub-Saharan Africa is still of low quality overall,^58^ and the historical data used in this study must be interpreted in the context of these limitations.

Our estimate of BL incidence in the period after ITN introduction was similar to the rate reported in a previous study (0.64 per 100 000 person-years in 2018).^59^ Since access to effective treatment for childhood cancer faces numerous challenges in sub-Saharan Africa and survival rates remain comparatively low,^60,61^ the possibility that BL incidence may be decreasing following successful malaria control programs has positive implications in terms of a reduced burden on patients and the health system. Given the higher political commitment to and funding of programs for infectious diseases, such as malaria and HIV infection, a multidisciplinary approach to addressing childhood cancer and infectious diseases has previously been suggested.^61,62^ Collaboration with existing malaria programs could raise awareness, attract funding, and increase political commitment to BL prevention, treatment, and research.^62^ Nevertheless, with plateauing funding and rising insecticide and treatment resistance, continued progress in malaria control also needs to remain a priority.

### Limitations

This study has several limitations. First, BL incidence rates were assembled from various locations, and only a subset of these had data at more than 1 time point. It is not known how representative these published data are of all incidence rates across locations in sub-Saharan Africa where malaria is endemic, and unknown systematic differences between the locations included in the period before ITN introduction compared with after could have biased the results. Second, our inclusion criteria were relatively broad to make use of all available data, but different methods in recording cancer incidence between locations and over time could have affected the results, especially as confirmation of diagnosis was frequently suboptimal (eTable 3 in Supplement 1). The small case numbers in some studies also suggest substantial underdiagnosis. Third, since an ecological study design was used, the association between ITN use and BL should not be interpreted to apply on the individual level; both ITN use and potential confounders only represent proxies for the average exposure in the population and were in many cases based on model estimates. The risk of ecological bias is particularly high because data were aggregated over large geographic areas, where spatial variability in the covariates is substantial. Fourth, we did not account for the use of other transmission-reducing malaria interventions (eg, treatment and indoor residual spraying), which could have confounded these results.

## Conclusions

The findings of this study support a continued focus on malaria control and suggest a potential additional benefit of ITNs in reducing the burden of one of the most common childhood cancers in sub-Saharan Africa. Analysis of all publicly available data on BL incidence in African countries where malaria is endemic accrued over almost 30 years indicated that incidence was lower after than before the introduction of mass ITN distribution programs around 2005. These findings are consistent with the existing observational evidence base for a potential role of malaria interventions in preventing BL in a context where conducting a randomized clinical trial would be unfeasible and unethical. However, data came from various sources and were often of low quality, underlining the need for improved cancer surveillance in sub-Saharan Africa to allow robust investigations of trends in cancer incidence.

## Notes

Supplement 1.
eMethods.
**eTable 1.** Search Strategy for Embase, Medline, and Global Health Databases **eFigure 1.** Directed Acyclic Graph for Model Covariate Selection for the Association Between Population-Level ITN Use and Burkitt Lymphoma Incidence **eFigure 2.** Estimated Insecticide-Treated Net Use Between 2000 and 2020 for Each Location With Data on Burkitt Lymphoma **eFigure 3.** Overview of Published Burkitt Lymphoma Incidence Rates Between 1990 and 2023 **eTable 2.** Overview of Included Data Points and Studies **eTable 3.** Quality Assessment of Included Studies From the Literature **eFigure 4.** Burkitt Lymphoma Incidence Rate in the Period Before and After Introduction of Insecticide-Treated Nets for Locations With at Least 1 Data Point in Each Period **eFigure 5.** Exposure-Response Relationship Between Insecticide-Treated Net Use and the Burkitt Lymphoma Incidence Rate in Sub-Saharan Africa in the Multivariate Model **eTable 4.** Sensitivity Analysis for Association Between Burkitt Lymphoma Incidence and ITN Use With Different ITN Use Exposure Periods **eTable 5.** Sensitivity Analysis for Association Between Burkitt Lymphoma Incidence and Insecticide-Treated Net Use **eTable 6.** Negative Binomial Regression Models for Association Between Burkitt Lymphoma Incidence and Absolute Reductions in Malaria Prevalence Over Time
eReferences

Supplement 2.
Data Sharing Statement
