# Supplementary material for: Mosquito Bed Net Use and Burkitt Lymphoma Incidence in Sub-Saharan Africa: A Systematic Review and Meta-Analysis
Source: JAMA Netw Open. 2024 Apr 18;7(4):e247351. doi: 10.1001/jamanetworkopen.2024.7351 (PMC12527478; doi:10.1001/jamanetworkopen.2024.7351)
Supplement: Supplement 1. — eMethods. eTable 1. Search Strategy for Embase, Medline, and Global Health Databases eFigure 1. Directed Acyclic Graph for Model Covariate Selection for the Association Between Population-Level ITN Use and Burkitt Lymphoma Incidence eFigure 2. Estimated Insecticide-Treated Net Use Between 2000 and 2020 for Each Location With Data on Burkitt Lymphoma eFigure 3. Overview of Published Burkitt Lymphoma Incidence Rates Between 1990 and 2023 eTable 2. Overview of Included Data Points and Studies eTable 3. Quality Assessment of Included Studies From the Literature eFigure 4. Burkitt Lymphoma Incidence Rate in the Period Before and After Introduction of Insecticide-Treated Nets for Locations With at Least 1 Data Point in Each Period eFigure 5. Exposure-Response Relationship Between Insecticide-Treated Net Use and the Burkitt Lymphoma Incidence Rate in Sub-Saharan Africa in the Multivariate Model eTable 4. Sensitivity Analysis for Association Between Burkitt Lymphoma Incidence and ITN Use With Different ITN Use Exposure Periods eTable 5. Sensitivity Analysis for Association Between Burkitt Lymphoma Incidence and Insecticide-Treated Net Use eTable 6. Negative Binomial Regression Models for Association Between Burkitt Lymphoma Incidence and Absolute Reductions in Malaria Prevalence Over Time eReferences [file jamanetwopen-e247351-s001.pdf]

Supplementary Online Content

Schmit N, Kaur J, Aglago EK. Mosquito bed net use and Burkitt lymphoma incidence in sub-Saharan Africa: a systematic review and meta-analysis. *JAMA Netw Open*. 2024;7(4):e247351. doi:10.1001/jamanetworkopen.2024.7351

eMethods.

eTable 1. Search Strategy for Embase, Medline, and Global Health Databases

eFigure 1. Directed Acyclic Graph for Model Covariate Selection for the Association Between Population-Level ITN Use and Burkitt Lymphoma Incidence

eFigure 2. Estimated Insecticide-Treated Net Use Between 2000 and 2020 for Each Location With Data on Burkitt Lymphoma

eFigure 3. Overview of Published Burkitt Lymphoma Incidence Rates Between 1990 and 2023

eTable 2. Overview of Included Data Points and Studies

eTable 3. Quality Assessment of Included Studies From the Literature

eFigure 4. Burkitt Lymphoma Incidence Rate in the Time Period Before and After Introduction of Insecticide-Treated Nets for Locations With at Least 1 Data Point in Each Period

eFigure 5. Exposure-Response Relationship Between Insecticide-Treated Net Use and the Burkitt Lymphoma Incidence Rate in Sub-Saharan Africa in the Multivariate Model

eTable 4. Sensitivity Analysis for Association Between Burkitt Lymphoma Incidence and ITN Use With Different ITN Use Exposure Periods

eTable 5. Sensitivity Analysis for Association Between Burkitt Lymphoma Incidence and Insecticide-Treated Net Use

eTable 6. Negative Binomial Regression Models for Association Between Burkitt Lymphoma Incidence and Absolute Reductions in Malaria Prevalence Over Time

eReferences.

This supplementary material has been provided by the authors to give readers additional information about their work.

© 2024 Schmit N et al. *JAMA Network Open*.

eMethods

Systematic review

Search strategy

eTable 1 shows the search terms for the systematic review conducted in the EMBASE, Medline and Global Health databases. We also searched for publications on websites associated with the International Agency for Research on Cancer.[1-3]

eTable 1. Search strategy for EMBASE, Medline and Global Health databases. The search strategy was developed and refined after cross-referencing of a pilot search against a list of known relevant papers. The original study protocol registered in PROSPERO (reference CRD42023411961) also included the African Journals Online database, but due to limitations in applying reproducible search strategies it was excluded.

**Database: EMBASE**	
1	"Africa south of the Sahara"/
2	(africa* or SSA or angola* or benin* or botswana* or burkina faso* or burundi* or cabo verd* or cameroon* or cameroun or cape verd* or central african republic* or chad* or comoros or (congo* not congo red) or cote divoir* or democratic republic of the congo* or djibouti* or equatorial guinea* or eritrea* or ethiopia* or gabon* or gambia* or ghan* or (guinea* not guinea pig* not guinea worm*) or guinea-bissau* or ivory coast or kenya* or lesotho* or liberia* or madagascar* or malawi* or mali or maurit* or mauritania* or mozambi* or namibia* or niger* or nigeria* or reunion or rwanda* or (sao tome and principe*) or senegal* or seychelle* or sierra leone* or somali* or south africa* or south sudan* or (sudan* not sudan blue) or swazi* or eswatini or tanzania* or togo* or uganda* or zambia* or zimbabwe*).mp. [mp=title, abstract, heading word, drug trade name, original title, device manufacturer, drug manufacturer, device trade name, keyword heading word, floating subheading word, candidate term word]
3	1 or 2
4	Burkitt lymphoma/
5	((burkitt* adj3 lymphoma*) or burkitt* lymphoma* or african lymphoma* or african malignant lymphoma* or burkitt* disease or burkitt* like lymphoma* or burkitt* tumor* or burkitt* tumour* or Lymphoma*, African or Lymphoma*, burkitt* or Malignant lymphoma*, African or non-hodgkin* lymphoma*).mp. [mp=title, abstract, heading word, drug trade name, original title, device manufacturer, drug manufacturer, device trade name, keyword heading word, floating subheading word, candidate term word]
6	4 or 5
7	(incidence or rate* or incident or distribution or pattern or prevalence or distribution or patterns or frequency).mp [mp=title, abstract, heading word, drug trade name, original title, device manufacturer, drug manufacturer, device trade name, keyword heading word, floating subheading word, candidate term word]
8	3 and 6 and 7
9	limit 8 to yr="1990 -Current"
**Database: Medline**	
1	"Africa South of the Sahara"/
2	(africa* or SSA or angola* or benin* or botswana* or burkina faso* or burundi* or cabo verd* or cameroon* or cameroun or cape verd* or central african republic* or chad* or comoros or (congo* not congo red) or cote divoir* or democratic republic of the congo* or djibouti* or equatorial guinea*

© 2024 Schmit N et al. *JAMA Network Open*.

	or eritrea* or ethiopia* or gabon* or gambia* or ghan* or (guinea* not guinea pig* not guinea worm*) or guinea-bissau* or ivory coast or kenya* or lesotho* or liberia* or madagascar* or malawi* or mali or maurit* or mauritania* or mozambi* or namibia* or niger* or nigeria* or reunion or rwanda* or (sao tome and principe*) or senegal* or seychelle* or sierra leone* or somali* or south africa* or south sudan* or (sudan* not sudan blue) or swazi* or eswatini or tanzania* or togo* or uganda* or zambia* or zimbabwe*).mp.
3	1 or 2
4	Burkitt lymphoma/
5	((burkitt* adj3 lymphoma*) or burkitt* lymphoma* or african lymphoma* or african malignant lymphoma* or burkitt* disease or burkitt* like lymphoma* or burkitt* tumor* or burkitt* tumour* or Lymphoma*, African or Lymphoma*, burkitt* or Malignant lymphoma*, African or non-hodgkin* lymphoma*).mp.
6	4 or 5
7	(incidence or rate* or incident or distribution or pattern or prevalence or distribution or patterns or frequency).mp. [mp=title, book title, abstract, original title, name of substance word, subject heading word, floating sub-heading word, keyword heading word, organism supplementary concept word, protocol supplementary concept word, rare disease supplementary concept word, unique identifier, synonyms, population supplementary concept word, anatomy supplementary concept word]
8	3 and 6 and 7
9	limit 8 to yr="1990 -Current"
**Database: Global Health**	
1	"Africa South of the Sahara"/
2	(africa* or SSA or angola* or benin* or botswana* or burkina faso* or burundi* or cabo verd* or cameroon* or cameroun or cape verd* or central african republic* or chad* or comoros or (congo* not congo red) or cote divoir* or democratic republic of the congo* or djibouti* or equatorial guinea* or eritrea* or ethiopia* or gabon* or gambia* or ghan* or (guinea* not guinea pig* not guinea worm*) or guinea-bissau* or ivory coast or kenya* or lesotho* or liberia* or madagascar* or malawi* or mali or maurit* or mauritania* or mozambi* or namibia* or niger* or nigeria* or reunion or rwanda* or (sao tome and principe*) or senegal* or seychelle* or sierra leone* or somali* or south africa* or south sudan* or (sudan* not sudan blue) or swazi* or eswatini or tanzania* or togo* or uganda* or zambia* or zimbabwe*).mp. [mp=abstract, title, original title, heading words, cabicodes words]
3	1 or 2
4	Burkitt's lymphoma.sh.
5	((burkitt* adj3 lymphoma*) or burkitt* lymphoma* or african lymphoma* or african malignant lymphoma* or burkitt* disease or burkitt* like lymphoma* or burkitt* tumor* or burkitt* tumour* or Lymphoma*, African or Lymphoma*, burkitt* or Malignant lymphoma*, African or non-hodgkin* lymphoma*).mp. [mp=abstract, title, original title, heading words, cabicodes words]
6	4 or 5
7	(incidence or rate* or incident or distribution or pattern or prevalence or distribution or patterns or frequency).mp. [mp=abstract, title, original title, heading words, cabicodes words]
8	3 and 6 and 7
9	limit 8 to yr="1990 -Current"

Inclusion and exclusion criteria

We included epidemiological studies reporting the incidence rate of BL in children aged 0-15 years in malaria-endemic sub-Saharan African countries. We applied no restrictions on study design, but

© 2024 Schmit N et al. *JAMA Network Open*.

modelling studies, such as those from the Global Burden of Disease Study, were excluded. Both population-based and hospital-based studies were considered for identification of cancer cases, but incidence rates had to apply to the general population. Therefore, we excluded populations selected for having specific known risk factors for Burkitt lymphoma (e.g. HIV-positive patients) or incidence rates calculated only among hospital patients. Populations residing outside of sub-Saharan Africa, e.g. immigrants of African origin, were also excluded. The outcome of interest was incident cancer cases diagnosed as BL in a defined population during a specified time period with a midpoint after 1990. Studies were eligible for inclusion if they reported the number of BL cases and either the person-time at risk or the crude incidence rate. Articles reporting only the frequency of Burkitt lymphoma cases without a defined catchment population at risk were excluded. Data on cancer cases was included if it was collected prospectively for the purpose of the study or extracted from medical records. Retrospective studies assessing cancer burden exclusively based on questionnaires administered to physicians were excluded.

Data processing

Where necessary, missing quantities (e.g. person-time at risk or incidence rate) were derived from the available information in the articles. Studies identified from the published literature were cross-checked against the dataset extracted from cancer registry publications for duplicates and overlap in data from the same registry. If case counts were sufficiently large (>10 in each period), we stratified exactly overlapping data from the same registry but two different sources into two separate shorter time periods. Data from two partially overlapping time periods were combined by calculating the mean incidence for the longer combined time period.

Quality assessment

For studies identified from the literature, quality was assessed based on three predefined criteria: the methods for data collection or cancer registration procedures, case ascertainment and diagnostic methods (including coding criteria), and calculation of person-time at risk. A score of 0 or 1 was assigned for each of these, and stated strengths and limitations were also considered. The total score was used to identify any particularly low-quality studies (score < 1). Quality assessment was not conducted on data extracted from IARC publications, as these all came from population-based cancer registries and had undergone a rigorous editorial process including quality control evaluation.[4,5] However, information on quality considerations for each registry was reviewed for notable deviations.

© 2024 Schmit N et al. *JAMA Network Open*.

Statistical analysis

Conceptual model

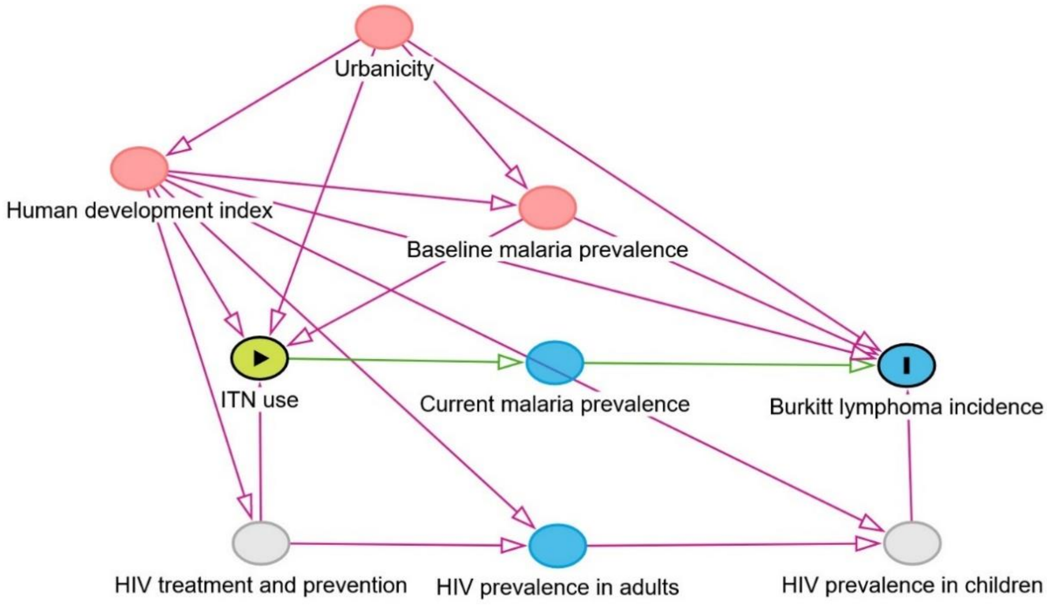

eFigure 1. Directed acyclic graph for model covariate selection for the association between population-level ITN use (exposure) and Burkitt lymphoma incidence (outcome). Green paths are causal while red paths are biasing, with red nodes indicating confounders. Grey nodes represent unobserved variables. The directed acyclic graph was created using DAGitty (https://www.dagitty.net/).

We used a directed acyclic graph for covariate selection in the model (

eFigure 1). We adjusted for the following potential confounders for the population-level relationship between ITN use and BL incidence: baseline malaria prevalence, human development index, urbanicity status of the population covered by the cancer registry or study, and concurrent HIV prevalence in adults.

Burkitt lymphoma primarily occurs in areas of high malaria transmission.[6,7] We hypothesised that population-wide ITN use could lead to reductions in Burkitt lymphoma incidence through its effect on reducing malaria transmission. Funding for malaria interventions is often targeted to the highest-transmission areas,[8,9] and baseline malaria prevalence in 2000, before large-scale implementation of interventions, therefore represents a potential confounder for the association between ITN use and Burkitt lymphoma incidence.

We also adjusted for the urban/rural status of a location as most population-based cancer registries are located in cities and cover urban populations,[4] while malaria is more common in rural areas.[10] We used the human development index as a metric of socioeconomic development – this is associated with urbanicity, and in turn may affect access to ITNs and malaria risk (e.g. through housing standards).[11] Measures of higher socioeconomic status were previously shown to be associated with reduced BL risk,[12] and the human development index correlates with cancer patterns.[13]

© 2024 Schmit N et al. *JAMA Network Open*.

We used HIV prevalence in adults as a proxy for HIV prevalence in children, for which no location-specific data could be found. HIV infection is a strong risk factor for Burkitt lymphoma outside of sub-Saharan Africa, including in patients treated with antiretroviral therapy.[14] Like malaria, HIV is associated with socioeconomic characteristics. HIV treatment programme scale-up also occurred in the 2000s and is supported by the same international funder as malaria.[15,16] We hypothesised that HIV prevalence might therefore also be associated with ITN use on the population level.[17]

Unit of analysis

Administrative divisions were based on the GADM database of Global Administrative Areas.[18] Catchments in the included studies had varying geographic bounds, covering either a first administrative level (admin1) unit, a subset of an admin1 unit or (subsets of) multiple admin1 units. For the analysis, we assumed the measured BL rate applied to the whole admin1 unit(s) in which data was collected. The corresponding average ITN use across these admin1 units was then estimated.

Regression model for the association between ITN use and BL incidence

We assumed that the observed number of cancer cases *y* follows a negative binomial distribution with mean μ and dispersion parameter θ, and accounted for clustering of datapoints collected in the same geographical location at different timepoints and for potential population-level confounders. The model is represented by the following set of equations:

$$y \sim NegBin(\mu, \theta)$$

$$\mu = exp(\beta_0 + \beta_1(ITN\ use) + \beta_2(PfPR2000) + \beta_3(HIV\ prevalence) + \beta_4(HDI\ level) + \beta_5(urbanicity) + \ln(\mathrm{pop}) + u)$$

$$u \sim N(0, \sigma_u^2)$$

$$\sigma^2 = \mu(1 + \frac{\mu}{\theta})$$

Where β_0 is the intercept, *β* parameters are the regression coefficients for the given fixed-effect predictor variables, *ln(pop)* is an offset to account for person-time at risk, and u is the location-specific random effect. σ^2 denotes the variance of the negative binomial distribution.

Software

The systematic review was conducted on the Covidence platform.[19] All analyses were conducted in R (v4.2.2).[20] ITN use estimates were extracted from the *foresite* package (v0.1.0).[21] The *glmmTMB* (v1.1.7) and *ggeffects* packages (v1.2.3) were used for the statistical analysis.[22,23]

© 2024 Schmit N et al. *JAMA Network Open*.

Supplementary Results

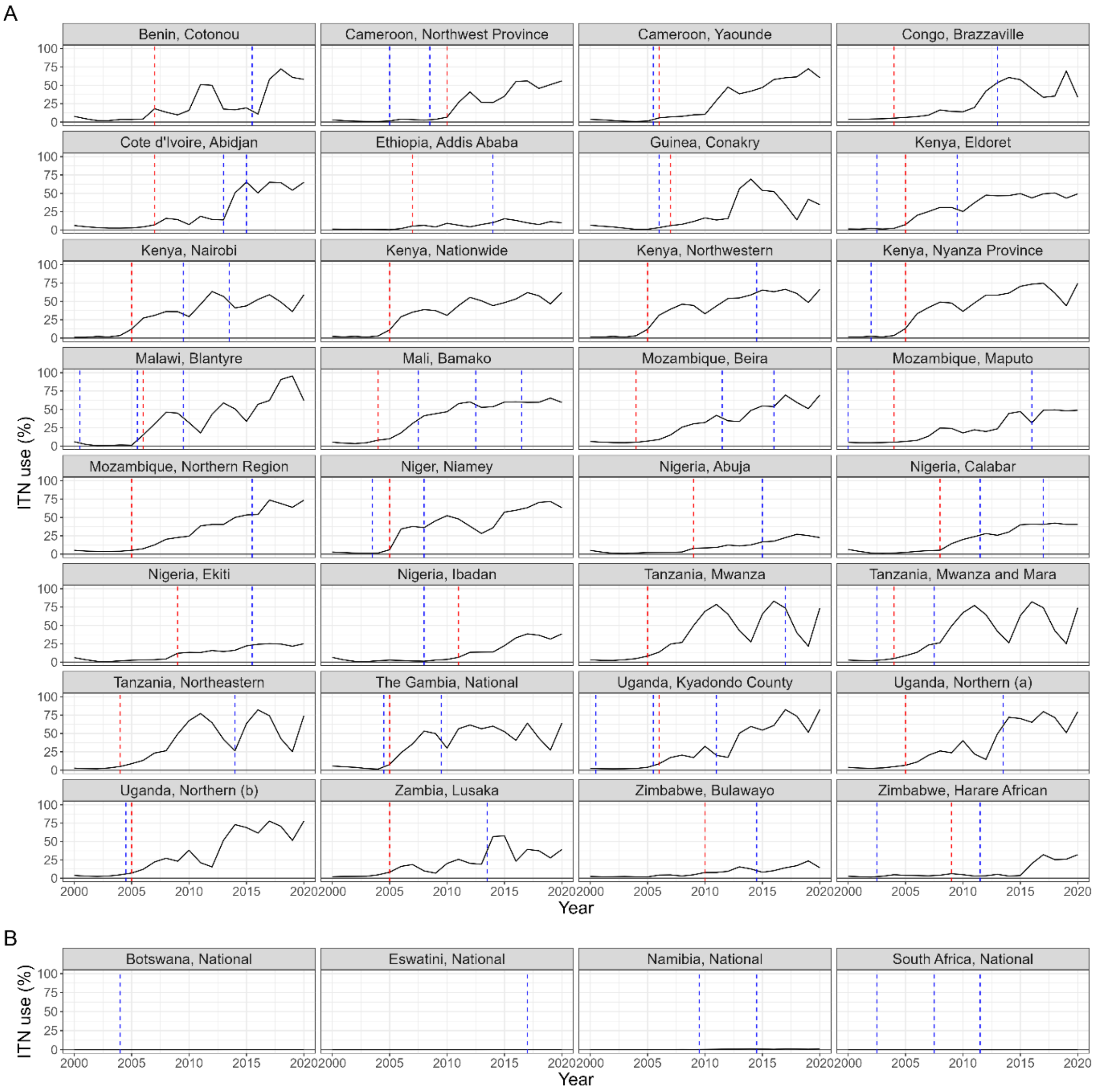

eFigure 2. Estimated insecticide-treated net (ITN) use between 2000 and 2020 for each location with data on Burkitt lymphoma. (A) Locations with large-scale population-wide ITN use. (B) Locations in southern Africa without large-scale ITN use (<1% in all years). Blue vertical lines represent the calendar midpoint for Burkitt lymphoma datapoints after 2000. Red vertical lines indicate the timepoint considered as the year of ITN introduction in the analysis.

© 2024 Schmit N et al. *JAMA Network Open*.

Among all included datapoints, 80% were obtained from population-based cancer registries (**eFigure 3A**). There was a moderate positive correlation between the mean reported BL incidence rate and the number of datapoints in a country, but no relationship between the number of datapoints and the HDI (**eFigure 3B**). BL incidence rates came from 32 locations in 21 countries, with 0-8 per country (**eFigure 3C**). Many countries in Central Africa had no data on BL incidence, while countries in East and Southern Africa had better coverage. The actual catchment area covered in these countries was usually very small (**eFigure 3D**). Only The Gambia in West Africa, and South Africa, Eswatini, Botswana and Namibia in Southern Africa had cancer registries with national-level coverage. However, these Southern African countries generally had the lowest BL rates. They were also considered separately, since analyses relating to ITN use were restricted to the 66 other datapoints from locations with large-scale ITN use, covering 17 countries.

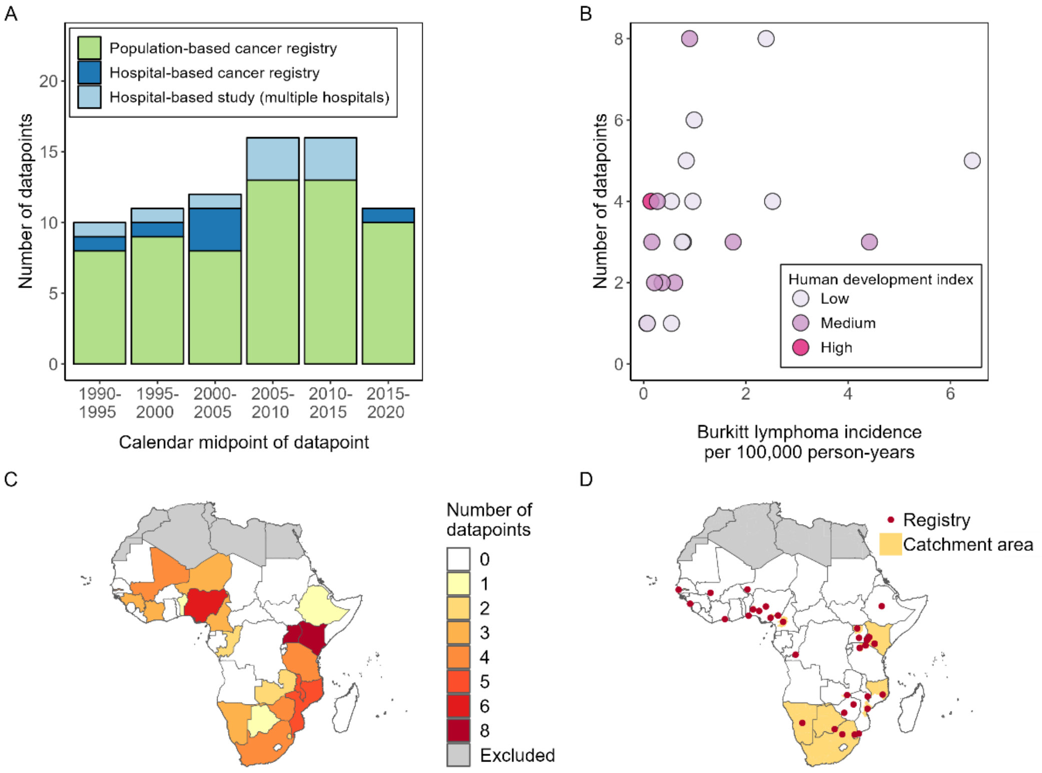

eFigure 3. Overview of published Burkitt lymphoma incidence rates between 1990 and 2023. Datapoints apply to different locations and/or different timepoints in a country. There were a total of 76 datapoints based on 5700 cases. (A) Number of datapoints by study type and over time. Cancer data was usually reported for periods of several years, but the number of studies were grouped based on the midpoint of each time period. (B) Number of datapoints by mean reported Burkitt lymphoma incidence rate in each country. Colours represent the human development index level of the country. (C) Number of datapoints in each country. (D) Location and catchment area of the covered population for each incidence datapoint. Yellow shading indicates the first administrative unit covered by the registry or study. Red points indicate the location of the registry or main referral hospital site for each datapoint.

© 2024 Schmit N et al. *JAMA Network Open*.

eTable *2*. Overview of included datapoints and studies. Some datapoints have multiple sources; this reflects that data for a specific time period in a cancer registry was derived by combining evidence from more than one publication.

Country	Location	Time period	Data source	Study type	Cases	Person-time at risk	Incidence rate per 100,000 person-years
Benin	Cotonou	2014-2016	Cancer in sub-Saharan Africa Vol 3	Population-based cancer registry	4	735900	0.54
Botswana	National	1999-2008	International Incidence of Childhood Cancer Vol 3 Cancer of Childhood in Africa	Population-based cancer registry	4	6334397	0.06
Cameroon	Northwest Province	2003-2006	Wright et al 2009	Hospital-based study (multiple)	44	745009	5.91
Cameroon	Northwest Province	2007-2009	Lewis et al 2012	Hospital-based study (multiple)	83	3149260	2.64
Cameroon	Yaounde	2004-2006	International Incidence of Childhood Cancer Vol 3	Population-based cancer registry	112	2381680	4.7
Congo	Brazzaville	1996-1999	Cancer in sub-Saharan Africa Vol 1	Population-based cancer registry	11	1017088	1.08
Congo	Brazzaville	2009-2016	Cancer in sub-Saharan Africa Vol 2<br>Cancer in sub-Saharan Africa Vol 3	Population-based cancer registry	6	4606275	0.13
Cote d'Ivoire	Abidjan	1995-1997	Cancer in sub-Saharan Africa Vol 1	Population-based cancer registry	49	3788100	1.29
Cote d'Ivoire	Abidjan	2012-2013	Cancer in sub-Saharan Africa Vol 2	Population-based cancer registry	41	3067686	1.34
Cote d'Ivoire	Abidjan	2014-2015	Cancer in sub-Saharan Africa Vol 3	Population-based cancer registry	77	2941106	2.62
Eswatini	National	1989-1999	Cancer in sub-Saharan Africa Vol 1	Population-based cancer registry	12	1656567	0.72
Eswatini	National	2016-2017	Cancer in sub-Saharan Africa Vol 3	Population-based cancer registry	0	828002	0
Ethiopia	Addis Ababa	2011-2016	Cancer of Childhood in Africa<br>Cancer in sub-Saharan Africa Vol 3	Population-based cancer registry	3	4207728	0.07
Guinea	Conakry	1993-1995	Cancer in sub-Saharan Africa Vol 1	Population-based cancer registry	10	1227118	0.81
Guinea	Conakry	1996-1999	Cancer in sub-Saharan Africa Vol 1	Population-based cancer registry	24	1983700	1.21
Guinea	Conakry	2001-2010	Cancer of Childhood in Africa	Population-based cancer registry	18	5818440	0.31
Kenya	Eldoret	1998-2006	International Incidence of Childhood Cancer Vol 3<br>Cancer of Childhood in Africa<br>Cancer in sub-Saharan Africa Vol 1	Population-based cancer registry	30	2774486	1.08
Kenya	Eldoret	2007-2011	Cancer of Childhood in Africa	Population-based cancer registry	11	1853425	0.59
Kenya	Nairobi	2007-2011	Cancer in sub-Saharan Africa Vol 2	Population-based cancer registry	18	4755015	0.38
Kenya	Nairobi	2012-2014	Cancer in sub-Saharan Africa Vol 3	Population-based cancer registry	12	3663807	0.33
Kenya	Nationwide	1988-1992	Mwanda et al 2004	Hospital-based study (multiple)	364	57284350	0.64

© 2024 Schmit N et al. *JAMA Network Open*.

Kenya	Nationwide	1993-1997	Mwanda et al 2004	Hospital-based study (multiple)	597	62210660	0.96
Kenya	Northwestern Kenya	2012-2016	Broen et al 2023	Hospital-based study (multiple)	183	17215428	1.06
Kenya	Nyanza Province	1999-2004	Rainey et al 2007	Hospital-based cancer registry	272	12648750	2.15
Malawi	Blantyre	1991-1995	International Incidence of Childhood Cancer Vol 2	Population-based cancer registry	54	1542334	3.5
Malawi	Blantyre	1996-1998	Banda et al 2001<br>International Incidence of Childhood Cancer Vol 2	Population-based cancer registry	18	975149	1.85
Malawi	Blantyre	1999-2001	Cancer in sub-Saharan Africa Vol 1<br>Banda et al 2001	Population-based cancer registry	23	1034687	2.22
Malawi	Blantyre	2003-2007	Cancer Incidence in Five Continents Vol X	Population-based cancer registry	308	1920229	16.04
Malawi	Blantyre	2008-2010	Cancer of Childhood in Africa<br>Cancer Incidence in Five Continents Vol X	Population-based cancer registry	102	1197708	8.52
Mali	Bamako	1988-1997	Cancer in sub-Saharan Africa Vol 1	Population-based cancer registry	3	3388015	0.09
Mali	Bamako	2005-2009	International Incidence of Childhood Cancer Vol 3 Cancer in sub-Saharan Africa Vol 2	Population-based cancer registry	80	3130224	2.56
Mali	Bamako	2010-2014	Cancer in sub-Saharan Africa Vol 2	Population-based cancer registry	16	4139565	0.39
Mali	Bamako	2015-2017	Cancer in sub-Saharan Africa Vol 3	Population-based cancer registry	25	3083991	0.81
Mozambique	Beira	2009-2013	Cancer in sub-Saharan Africa Vol 2	Population-based cancer registry	9	875560	1.03
Mozambique	Beira	2014-2017	Cancer in sub-Saharan Africa Vol 3	Population-based cancer registry	5	680788	0.73
Mozambique	Maputo	1991-2008	Lorenzoni et al 2015	Hospital-based cancer registry	133	7130923	1.87
Mozambique	Maputo	2014-2017	Lorenzoni et al 2020	Population-based cancer registry	2	1387948	0.14
Mozambique	Northern Region	2015-2015	O'Callaghan-Gordo et al 2016	Hospital-based cancer registry	19	4786000	0.4
Namibia	National	1995-1998	Cancer in sub-Saharan Africa Vol 1	Population-based cancer registry	3	2683828	0.11
Namibia	National	2009-2009	Cancer in sub-Saharan Africa Vol 2	Population-based cancer registry	0	818705	0
Namibia	National	2013-2015	Cancer in sub-Saharan Africa Vol 3	Population-based cancer registry	9	2443227	0.37
Niger	Niamey	1993-1999	Cancer in sub-Saharan Africa Vol 1	Population-based cancer registry	11	1744519	0.63
Niger	Niamey	2001-2005	Cancer of Childhood in Africa<br>Cancer in sub-Saharan Africa Vol 2	Population-based cancer registry	18	1621670	1.11
Niger	Niamey	2006-2009	Cancer in sub-Saharan Africa Vol 2	Population-based cancer registry	9	1717564	0.52
Nigeria	Abuja	2013-2016	Cancer in sub-Saharan Africa Vol 3	Population-based cancer registry	0	2373848	0

© 2024 Schmit N et al. *JAMA Network Open*.

Nigeria	Calabar	2009-2013	Cancer in sub-Saharan Africa Vol 2	Population-based cancer registry	9	629535	1.43
Nigeria	Calabar	2016-2017	Cancer in sub-Saharan Africa Vol 3	Population-based cancer registry	1	341324	0.29
Nigeria	Ekiti	2013-2017	Cancer in sub-Saharan Africa Vol 3	Population-based cancer registry	3	1081990	0.28
Nigeria	Ibadan	1993-1999	Cancer in sub-Saharan Africa Vol 1	Population-based cancer registry	39	1607882	2.43
Nigeria	Ibadan	2006-2009	Cancer in sub-Saharan Africa Vol 2	Population-based cancer registry	29	1927064	1.5
South Africa	National	1989-1992	Cancer in sub-Saharan Africa Vol 1	Population-based cancer registry	45	51416511	0.09
South Africa	National	1998-2006	International Incidence of Childhood Cancer Vol 3 Cancer in sub-Saharan Africa Vol 2 Cancer of Childhood in Africa	Population-based cancer registry	201	1.29E+08	0.16
South Africa	National	2007-2007	Cancer in sub-Saharan Africa Vol 2	Population-based cancer registry	24	15010300	0.16
South Africa	National	2008-2014	Cancer of Childhood in Africa<br>Cancer in sub-Saharan Africa Vol 3	Population-based cancer registry	176	1.07E+08	0.16
Tanzania	Mwanza	2016-2017	Cancer in sub-Saharan Africa Vol 3	Population-based cancer registry	6	642170	0.93
Tanzania	Mwanza and Mara	2000-2004	Aka et al 2012	Hospital-based study (multiple)	540	10114350	5.34
Tanzania	Mwanza and Mara	2005-2009	Aka et al 2012	Hospital-based study (multiple)	404	11735304	3.44
Tanzania	Mwanza and Mara	2012-2015	Broen et al 2023	Hospital-based study (multiple)	94	25410153	0.37
The Gambia	National	1988-1996	Cancer in sub-Saharan Africa Vol 1<br>Cancer Incidence in Five Continents Vol VIII	Population-based cancer registry	19	3667934	0.52
The Gambia	National	1997-1998	Cancer Incidence in Five Continents Vol VIII	Population-based cancer registry	10	994516	1.01
The Gambia	National	2002-2006	Cancer of Childhood in Africa<br>Cancer in sub-Saharan Africa Vol 2	Population-based cancer registry	16	3302980	0.48
The Gambia	National	2007-2011	Cancer in sub-Saharan Africa Vol 2	Population-based cancer registry	6	3723130	0.16
Uganda	Kyadondo County	1989-1991	Wabinga et al 1993	Population-based cancer registry	24	1305684	1.84
Uganda	Kyadondo County	1993-1997	Cancer Incidence in Five Continents Vol VIII	Population-based cancer registry	90	2400040	3.75
Uganda	Kyadondo County	1998-2002	Cancer Incidence in Five Continents Vol IX	Population-based cancer registry	64	3068310	2.09
Uganda	Kyadondo County	2003-2007	Cancer Incidence in Five Continents Vol X	Population-based cancer registry	158	3767734	4.19
Uganda	Kyadondo County	2008-2013	Cancer Incidence in Five Continents Vol XI<br>Cancer in sub-Saharan Africa Vol 3	Population-based cancer registry	92	5395883	1.71

© 2024 Schmit N et al. *JAMA Network Open*.

Uganda	Northern Uganda	2010-2016	Broen et al 2023	Hospital-based study (multiple)	267	13471241	1.98
Uganda	Northern Uganda	1997-2001	Ogwang et al 2008	Hospital-based cancer registry	132	13888889	0.95
Uganda	Northern Uganda	2002-2006	Ogwang et al 2008	Hospital-based cancer registry	368	13888889	2.65
Zambia	National	1990-1992	Chintu et al 1995	Hospital-based cancer registry	11	9166667	0.12
Zambia	Lusaka	2011-2015	Cancer in sub-Saharan Africa Vol 3	Population-based cancer registry	13	4246505	0.31
Zimbabwe	Bulawayo	2013-2015	Cancer in sub-Saharan Africa Vol 3	Population-based cancer registry	4	754257	0.53
Zimbabwe	Harare	1990-1997	Cancer in sub-Saharan Africa Vol 1	Population-based cancer registry	6	3435108	0.17
Zimbabwe	Harare	1998-2006	Cancer Incidence in Five Continents Vol IX<br>Cancer Incidence in Five Continents Vol X	Population-based cancer registry	9	4873482	0.18
Zimbabwe	Harare	2007-2015	Cancer of Childhood in Africa<br>Cancer Incidence in Five Continents Vol X<br>Cancer in sub-Saharan Africa Vol 3	Population-based cancer registry	8	4427652	0.18

© 2024 Schmit N et al. *JAMA Network Open*.

eTable 3. **Quality assessment of included studies from the literature.** The total score is the sum of individual scores in each column, with a possible range from -1 (highest risk of bias) to 4 (lowest risk of bias). BL = Burkitt lymphoma.

Study	Type of study	Data collection [score]	Confirmation of diagnosis [score]	Calculation of person-time at risk [score]	Notable stated strengths and limitations [score]	Score
Aka et al., 2012, Pediatric Blood & Cancer	Hospital-based study (multiple hospitals)	Case information compiled by a research assistant from routine hospital records from 6 hospitals in Mara and Mwanza regions. The hospitals included were all those able to diagnose and treat BL in the regions. Only cases with a home address in the Mara and Mwanza regions were included. Cases from these two regions that may have been treated at a hospital outside the region were not searched for. [1]	Predominantly clinical diagnosis with confirmation using fine needle aspiration in a few cases. [0]	Annual age-specific population size was extrapolated forward and backwards from the 2002 national census according to region-specific growth rates. [1]	NA [0]	2
Wright et al., 2009, Tropical Doctor	Hospital-based study (multiple hospitals)	Case data collected from all 16 hospitals, private clinics, the regional pathologist and the Delegation of Public Health in the Northwest province. Only cases in 2015 were included as it was the only year where all hospitals provided data on BL. [1]	Cases were included if they had either a histological diagnosis (70%) or a clinical diagnosis of a fast-growing tumour, that responded to the Malawi 2002 protocol with complete regression within six chemotherapy doses, or, if they died before, an initial response of >50% reduction within three doses. [1]	Population data provided by the Delegation of Public Health, no further information. [1]	NA [0]	3
Ogwang et al., 2008, International Journal of Cancer	Hospital-based cancer registry	Case data collected in hospital-based cancer registry of the only referral hospital in the region with facilities to both diagnose and treat BL. Only cases with an address in the 10 neighbouring districts were included, for a time period where registry data was considered reasonably complete. [0]	Cases are diagnosed clinically and confirmed using cytology or histology by a senior pathologist at Makerere University Medical School in Kampala. [1]	Annual age-specific population projections were obtained from the Uganda Bureau of Statistics, which were based on population counts from the 1991 and 2002 censuses. [1]	Civil unrest in 1997-2001. Data patterns suggest underreporting (e.g. lower incidence in counties further from Lacor especially during period of civil unrest). [-1]	1
Broen et al., 2023, Proceedings of the National	Hospital-based study (multiple hospitals, population-	BL cases were recruited at 6 local district or regional hospitals serving a population living in defined geographic areas (2 regions in each country). Only cases who were usual residents (≥4 months prior to	Cases were defined histologically or cytologically (61%), and when this was not possible, according to clinical features, imaging and laboratory	Age-specific population data obtained from each country's statistical bureau and interpolated using district or regional average population	Fulltime field staff were hired to implement the study in the three countries and trained by the	3

© 2024 Schmit N et al. *JAMA Network Open*.

Academy of Sciences of the United States of America	based case control study)	enrollment) of the study area were included. To increase ascertainment and encourage referral of suspected cases to the six participating hospitals, health education messages about BL were developed and disseminated in the study area. [1]	results compatible with a diagnosis of BL. [1]	growth rates. National census data was available from 2009 in Kenya and 2002 and 2014 in Uganda. [1]	same instructors at Makerere University College of Health Sciences in Uganda. [0]	
Lorenzoni et al., 2020, International Journal of Cancer	Population-based cancer registry	Case data collected from 3 hospitals (including the national referral hospital responsible for most cancer cases diagnoses and registrations), the Private Laboratory of Pathology and death certificates. Death registration is mandatory and death certificates are completed by a physician (for hospital deaths) or the statistical office in the mortuary (for deaths at home). For deaths at home, cause of death information is based on documents brought by the family, and questions to them similar to verbal autopsy. Deaths from cancer are matched with the registry database and hospital records. Only cases resident in Maputo City were included. The Canreg5 system for deduplication, data management and analysis was used. [1]	Tumour site and histology coded according to ICD-O-3 and converted to ICD-10 for analysis. 74% of all non-Hodgin lymphoma diagnoses were morphologically verified and 24% were based on death certificates only. [1]	Annual age-specific population size was interpolated based on the the census of 2007 and 2017 using a constant age- and sex-specific growth rate. [1]	NA [0]	3
O'Callaghan-Gordo et al., 2016, The American Journal of Tropical Medicine and Hygiene	Hospital-based cancer registry	Prospective collection of all new BL diagnoses in Nampula Central Hospital, which is the referral hospital and has the only pathology department in the region. Only cases living in the region were included. [0]	Diagnosis was confirmed using fine-needle aspiration cytology (82%) or histology (18%) in all but 2 cases. Diagnosis was confirmed by two pathologists. [1]	Age-specific population estimates were obtained from the official projections of the National Institute of Statistics of Mozambique, based on data from the censuses conducted in 1997 and 2007. [0]	NA [0]	1
Lewis et al., 2012, Paediatrics and	Hospital-based study (multiple hospitals)	Case data collected in the three treatment centers for BL in the country by a specialist BL nurse and a standardised inpatient work-up. Cases	Diagnosis confirmed by fine needle aspiration or based on strict clinical criteria pertaining to a fast growing	Age-specific population size in the 2007 census obtained from the Delegation of Public Health	In Northwest Province, the two BL treatment centres have a long-standing	4

© 2024 Schmit N et al. *JAMA Network Open*.

International Child Health		were classified according to their home district. [1]	tumour responding to BL treatment. [1]	and extrapolated using a constant growth rate. [1]	history, a well established education programme for clinicians and patients and provide treatment free of charge. This suggests referral from local health centers is likely. [1]	
Rainey et al., 2007, International Journal of Cancer	Hospital-based cancer registry	Case data collected from medical records of all BL cases admitted to Nyanza Provincial General Hospital (the referral center for childhood cancer cases and only treatment center for BL in the region) with a physician diagnosis of Burkitt's lymphoma. Only cases residing in Nyanza Province were included and duplicate cancer records were excluded. [0]	All diagnoses histologically confirmed. For cases with incomplete histology records, inclusion was restricted to those treated for BL and responding to treatment with no competing diagnosis. [1]	Age-specific population estimates based on 1999 census data obtained from the Kenya Medical Research Institute/Wellcome Trust Collaborative Programme and 2000–2004 age-specific population projections generated by the Kenyan Central Bureau of Statistics. [1]	Cancer cases from isolated communities were captured in the hospital records, suggesting that the majority of cases in the region were detected. [0]	2
Banda et al., 2001, Tropical Medicine & International Health	Population-based cancer registry	Case data collected using active and passive methods of case-finding, including: reports on all cancer cases diagnosed in the central pathology laboratory at Queen Elizabeth Central Hospital (the only one in the country), visits to inpatient wards and oncology outpatient clinics of the hospital and seven other hospitals in the district (including private hospitals), and searching of the register of cancer patients who die in Queen Elizabeth Central Hospital. Only cases resident in Blantyre Districts for at least 6 months were included. The Canreg system for deduplication, data management and analysis was used. [1]	Tumour site and histology coded according to ICD-O-2 and converted to ICD-10 for analysis. 77% of all childhood cancer cases were morphologically verified. [1]	Age-specific population size was interpolated between census data from 1987 and 1998. [1]	NA [0]	3
Chintu et al., 1995, Archives of	Hospital-based cancer registry	Case data collected from all histopathological records at the University Teaching Hospital, which is	All diagnoses were based on histopathology. [1]	Age-specific population size in the whole country was obtained	NA [0]	1

© 2024 Schmit N et al. *JAMA Network Open*.

Disease in Childhood		estimated to cover 77% of the total paediatric population of Zambia. Duplicates and cases with "doubtful information" on diagnosis were excluded. [0]		from projections based on the 1980 and 1990 census. [0]		
Wabinga et al., 1993, International Journal of Cancer	Population-based cancer registry	Case data collected from histopathological records from the only pathology service in Uganda with diagnostic histological, cytological and autopsy capacity, and active case finding through monthly visits by the cancer registrar to the 4 major hospitals in Uganda to which cancer cases might be admitted (Mulago, Mengo, Rubaga and Nsambya). Only cases resident in Kyadondo County for at least 1 year were included. [1]	Tumour site and histology coded according to ICD-O and converted to ICD-9 for analysis. 71% of all lymphoma cases were confirmed histologically/cytologically and 26% were based solely on clinical examination. [1[	Age-specific population size assumed to be the same in each year as in the 1991 census. [1]	NA [0]	3
Lorenzoni et al., 2015, PLoS One	Hospital-based cancer registry	Case data collected from all cancer cases registered in the Department of Pathology of the Maputo Central Hospital, which is the national referral hospital. Cases from sites other than Maputo and duplicate registrations were excluded. [0]	Tumour site and histology coded according to ICD-O and converted to ICD-10 for analysis. All diagnoses were based on histopathology. [1]	Age-specific population size was interpolated between national censuses in 1980, 1997 and 2007 assuming a constant sex- and age-specific growth rate. [1]	NA [0]	2
Mwanda et al., 2004, East African Medical Journal	Hospital-based study (multiple hospitals)	Case data collected from seven provincial hospitals (Coast General Hospital, Garissa, Embu, Nyeri, Nakuru, Kakamega, Kisumu) and the national referral hospital with BL treatment facilities (Kenyatta National Hospital). Medical records were reviewed between 1988-1992 and data was collected prospectively between 1993-1997. Duplicate registrations were excluded and residence was assigned based on the previous 3 months. [1]	Diagnosis was primarily histological using fine needle aspiration and corroborated by clincial presentation and treatment results. [1]	Age-specific population size estimated based on census data provided by Kenya National Population Census Offices (no further information). [1]	Difficulty in ascertaining residence, particularly in Nairobi, and questions regarding denominator quality. Better case ascertainment in the prospective than the retrospective period. [-1]	2

© 2024 Schmit N et al. *JAMA Network Open*.

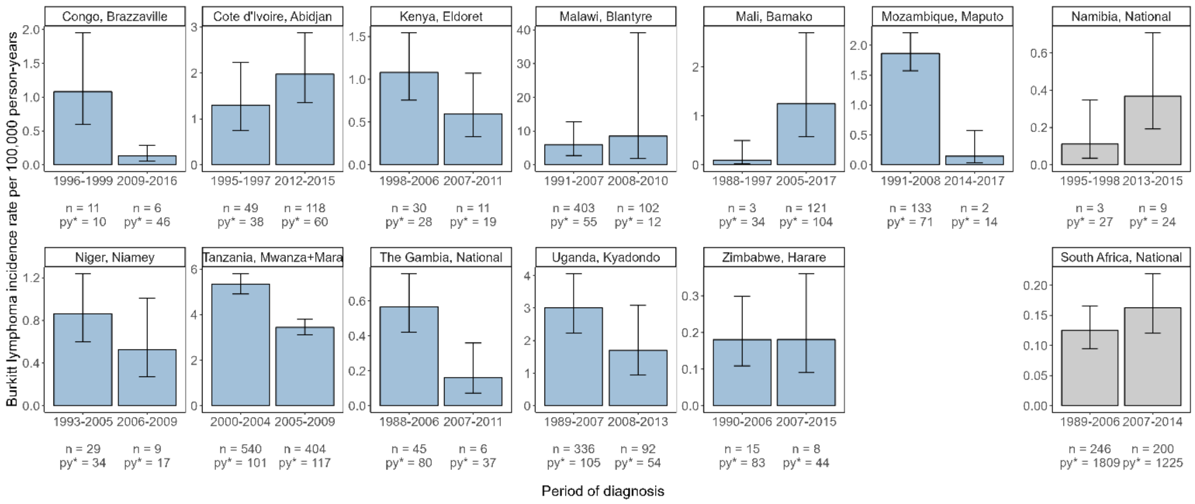

eFigure 4. **Burkitt lymphoma incidence rate in the time period before and after introduction of insecticide-treated nets (ITNs) for locations with at least one datapoint in each period.** Namibia and South Africa (grey bars) did not have wide-scale ITN use but were separated into two time periods with midpoints before and after 2005. Incidence rates with 95% confidence intervals were calculated using a negative binomial model and adjusted for clustering in data from the same geographical location. The number of cases (n) and person-years at risk in 100,000s (py*) are given for each time period. The indicated period of diagnosis represents the minimum and maximum year covered in the dataset but may not be fully covered by data.

© 2024 Schmit N et al. *JAMA Network Open*.

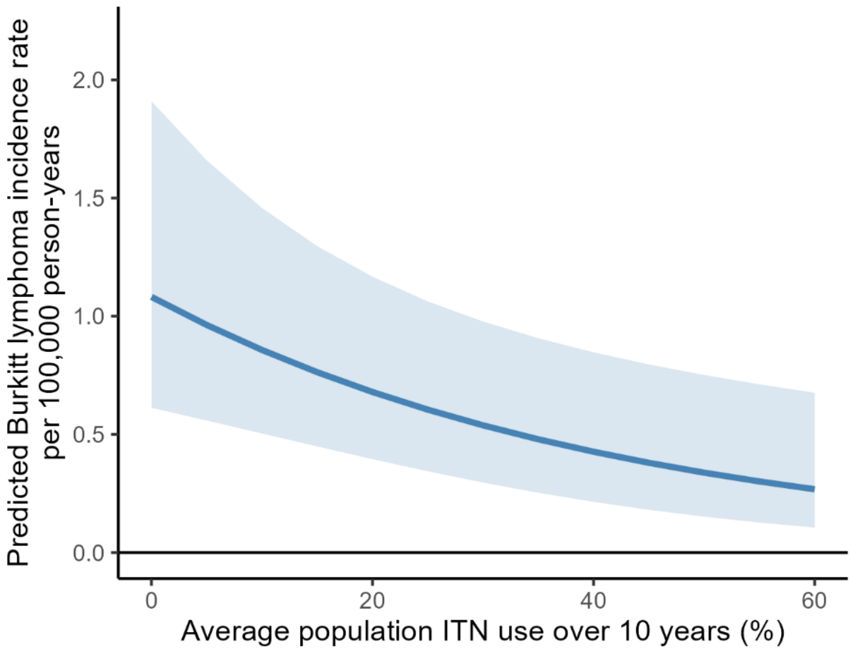

eFigure 5. Exposure-response relationship between insecticide-treated net (ITN) use and the Burkitt lymphoma incidence rate in sub-Saharan Africa in the multivariate model. ITN use represents the mean ITN use in the population at the first administrative level in the 10 years before the Burkitt lymphoma data collection period.

© 2024 Schmit N et al. *JAMA Network Open*.

Sensitivity analyses

Comparing different exposure time periods for ITN use, the association was similar for average ITN use in the 5 and 15 years before the BL datapoint [rate ratio 0.98 (95% CI 0.97-0.99) and 0.97 (95% CI 0.95-0.99), respectively], while concurrent ITN use in the population was not strongly associated with BL incidence (rate ratio 0.99, 95% CI 0.98-1.00) (**eTable 5**). Removing influential outlier studies and restricting the analysis to locations with BL incidence data before and after ITN introduction did not affect conclusions about the association between ITN use and BL incidence, though the strength of the association was lower in some cases (**eTable 6**). Similarly, a 1 percentage point decrease in the mean malaria prevalence in the 10 years before the Burkitt lymphoma data collection period compared to the baseline prevalence in 2000 was associated with a 4% (95% CI 1-6%) reduction in BL incidence (**eTable 7**).

© 2024 Schmit N et al. *JAMA Network Open*.

eTable 4. **Sensitivity analysis for association between Burkitt lymphoma incidence and insecticide-treated net (ITN) use with different ITN use exposure periods.**

	ITN use predictor: Average population ITN use over 5 years		ITN use predictor: Average population ITN use over 15 years		ITN use predictor: Concurrent ITN use	
**Covariate**	**Adjusted rate ratio (95% CI)**	**p-value**	**Adjusted rate ratio (95% CI)**	**p-value**	**Adjusted rate ratio (95% CI)**	**p-value**
**ITN use predictor (%)**	0.98 (0.97-0.99)	0.005	0.97 (0.95-0.99)	0.005	0.99 (0.98-1.00)	0.09
**Baseline malaria parasite prevalence in 2000 (%)**	1.02 (1.01-1.04)	0.01	1.02 (1.01-1.04)	0.01	1.02 (1.01-1.04)	0.01
**HIV prevalence in 15-49 year olds (%)**	1.01 (0.96-1.07)	0.67	1.01 (0.96-1.07)	0.60	1.02 (0.97-1.08)	0.47
**Country Human Development Index level**						
HDI < 0.45	1.00 (reference)		1.00 (reference)		1.00 (reference)	
HDI ≥ 0.45	0.97 (0.57-1.65)	0.91	0.95 (0.56-1.61)	0.85	0.92 (0.53-1.58)	0.75
**Population urbanicity status**						
Urban	1.00 (reference)		1.00 (reference)		1.00 (reference)	
Both	1.24 (0.60-2.57)	0.57	1.24 (0.60-2.57)	0.56	1.23 (0.60-2.55)	0.57
Rural	2.05 (0.61-6.85)	0.24	2.11 (0.64-6.97)	0.22	2.23 (0.68-7.28)	0.18

© 2024 Schmit N et al. *JAMA Network Open*.

eTable 2. Sensitivity analysis for association between Burkitt lymphoma incidence and insecticide-treated net (ITN) use. n indicates the number of incidence datapoints included in each model. Models 1, 2 and 3 included 4282, 5226 and 2473 cancer cases, respectively. The influential outliers, based on a Cook's distance of greater than 4/(total number of studies), were Burkitt lymphoma incidence in Mara and Mwanza, Tanzania, 2000-2009[24] and Abuja population-based cancer registry, Nigeria, 2013-2016.[4] The study set in Tanzania contributed 17% of all cancer cases in the dataset and, while not among the lowest-scoring studies for quality overall (eTable 3), was the only one where diagnosis was almost exclusively based on clinical assessment. Abuja cancer registry recorded zero Burkitt lymphoma cases, and quality concerns about the data from this registry were raised in the publication.

	**Model 1: Remove influential outlier 1 (n = 64)**		**Model 2: Remove influential outlier 2 (n = 65)**		**Model 3: Only locations with BL data before and after ITN introduction (n = 35)**	
**Covariate**	**Adjusted rate ratio (95% CI)**	**p-value**	**Adjusted rate ratio (95% CI)**	**p-value**	**Adjusted rate ratio (95% CI)**	**p-value**
**Average population ITN use over 10 years (%)**	0.98 (0.97- 1.00)	0.06	0.98 (0.96-0.99)	0.002	0.97 (0.95-1.00)	0.05
**Baseline malaria parasite prevalence in 2000 (%)**	1.03 (1.01- 1.05)	0.002	1.03 (1.01-1.05)	0.001	1.04 (1.01-1.07)	0.01
**HIV prevalence in 15-49 year olds (%)**	1.02 (0.97- 1.08)	0.40	1.01 (0.96-1.07)	0.59	0.98 (0.93-1.04)	0.52
**Country Human Development Index level**						
HDI < 0.45	1.00 (reference)		1.00 (reference)		1.00 (reference)	
HDI ≥ 0.45	1.05 (0.61-1.82)	0.85	1.05 (0.63-1.74)	0.86	0.82 (0.43-1.55)	0.54
**Population urbanicity status**						
Urban	1.00 (reference)		1.00 (reference)		1.00 (reference)	
Both	1.02 (0.50- 2.08)	0.95	1.04 (0.51-2.11)	0.91	3.28 (1.32-8.16)	0.01
Rural	2.34 (0.76-7.21)	0.14	2.02 (0.64-6.38)	0.23	0.80 (0.18-3.55)	0.76

© 2024 Schmit N et al. *JAMA Network Open*.

eTable 3. Negative binomial regression models for association between Burkitt lymphoma incidence and absolute reductions in malaria prevalence over time. The reduction in malaria prevalence in 2-10 year olds was calculated as the mean absolute reduction in the 10 years before the Burkitt lymphoma data collection period compared to the baseline prevalence in 2000 (representing a percentage point decrease). The analysis was conducted on all 76 Burkitt lymphoma incidence estimates.

Covariate	Crude rate ratio (95% CI)	*p*-value	Adjusted rate ratio (95% CI)	*p*-value
**Average absolute reduction in 10-year malaria prevalence compared to 2000 baseline (%)**	0.98 (0.96-1.00)	0.09	0.96 (0.94-0.99)	0.006
**Baseline malaria parasite prevalence in 2000 (%)**	1.03 (1.01-1.05)	0.0005	1.04 (1.02-1.06)	0.00001
**HIV prevalence in 15-49 year olds (%)**	0.98 (0.93-1.03)	0.44	1.01 (0.96-1.05)	0.83
**Country Human Development Index level**				
HDI < 0.45	1.00 (reference)		1.00 (reference)	
HDI ≥ 0.45	0.66 (0.38-1.14)	0.14	0.84 (0.50-1.43)	0.52
**Population urbanicity status**				
Urban	1.00 (reference)		1.00 (reference)	
Both	1.04 (0.45-2.40)	0.92	0.97 (0.51-1.84)	0.92
Rural	2.05 (0.41-10.26)	0.38	1.96 (0.60-6.40)	0.26

© 2024 Schmit N et al. *JAMA Network Open*.

eReferences

1. International Agency for Research on Cancer. IARC Scientific Publications. February 27, 2023, Accessed February 27, 2023, https://publications.iarc.fr/
2. International Agency for Research on Cancer. Global Cancer Observatory. February 27, 2023, February 27, 2023. https://gco.iarc.fr/
3. African Cancer Registry Network. AFCRN Publications. February 27, 2023, February 27, 2023. https://afcrn.org/
4. Parkin DM, Jemal A, Bray F, et al, eds. *Cancer in sub-Saharan Africa Vol 3*. Union for International Cancer Control; 2019.
5. Bray F, Colombet M, Mery L, et al, eds. *Cancer Incidence in Five Continents Vol XI. IARC Scientific Publication No 166*. International Agency for Research on Cancer; 2021.
6. Gondwe Y, Salima A, Manda A, et al. Spatial distribution of incident pediatric Burkitt lymphoma in central and northern Malawi and association with malaria prevalence. *Pediatr Blood Cancer*. Oct 2022;69(10):e29867. doi:10.1002/pbc.29867
7. Broen K, Dickens J, Trangucci R, et al. Burkitt lymphoma risk shows geographic and temporal associations with Plasmodium falciparum infections in Uganda, Tanzania, and Kenya. *Proc Natl Acad Sci U S A*. Jan 10 2023;120(2):e2211055120. doi:10.1073/pnas.2211055120
8. The Global Fund. Description of the 2020-2022 Allocation Methodology. Accessed August 30, 2023, https://www.theglobalfund.org/media/9224/fundingmodel_2020-2022allocations_methodology_en.pdf
9. Wetzler EA, Park C, Arroz JA, Chande M, Mussambala F, Candrinho B. Impact of mass distribution of insecticide-treated nets in Mozambique, 2012 to 2025: Estimates of child lives saved using the Lives Saved Tool. *PLOS Global Public Health*. 2022;2(4):e0000248.
10. World Health Organization. *World malaria report 2022*. 2022. 9240064893.
11. Kim H, Miller FD, Hernandez A, Tanser F, Mogeni P, Cuadros DF. Spatiotemporal analysis of insecticide-treated net use for children under 5 in relation to socioeconomic gradients in Central and East Africa. *Malaria journal*. 2020;19(1):1-16.
12. Peprah S, Ogwang MD, Kerchan P, et al. Risk factors for Burkitt lymphoma in East African children and minors: A case-control study in malaria-endemic regions in Uganda, Tanzania and Kenya. *Int J Cancer*. Feb 15 2020;146(4):953-969. doi:10.1002/ijc.32390
13. Sharma R, Aashima, Nanda M, et al. Mapping Cancer in Africa: A Comprehensive and Comparable Characterization of 34 Cancer Types Using Estimates From GLOBOCAN 2020. *Front Public Health*. 2022;10:839835. doi:10.3389/fpubh.2022.839835
14. Atallah-Yunes SA, Murphy DJ, Noy A. HIV-associated Burkitt lymphoma. *The Lancet Haematology*. 2020;7(8):e594-e600.
15. GBD 2017 HIV collaborators. Global, regional, and national incidence, prevalence, and mortality of HIV, 1980-2017, and forecasts to 2030, for 195 countries and territories: a systematic analysis for the Global Burden of Diseases, Injuries, and Risk Factors Study 2017. *Lancet HIV*. Dec 2019;6(12):e831-e859. doi:10.1016/s2352-3018(19)30196-1
16. World Health Organization. *World malaria report 2020*. 2020.
17. Alene KA, Elagali A, Barth DD, et al. Spatial codistribution of HIV, tuberculosis and malaria in Ethiopia. *BMJ Global Health*. 2022;7(2):e007599.
18. GADM. Maps and data, v4.04. Accessed Aug 3, 2023, https://gadm.org/
19. Veritas Health Innovation. Covidence systematic review software. www.covidence.org
20. R Core Team. R: A language and environment for statistical computing. R Foundation for Statistical Computing. https://www.R-project.org/
21. Winskill P. foresite: Access to Malaria Site Files. R package version 0.1.0. https://mrc-ide.github.io/foresite/
22. Magnusson A, Skaug HJ, Nielsen A, et al. glmmTMB: Generalized Linear Mixed Models using Template Model Builder. R package version 0.1.3. https://github.com/glmmTMB

23. Lüdecke D. ggeffects: Tidy data frames of marginal effects from regression models. *Journal of Open Source Software*. 2018;3(26):772.
24. Aka P, Kawira E, Masalu N, et al. Incidence and trends in Burkitt lymphoma in northern Tanzania from 2000 to 2009. *Pediatric blood & cancer*. 2012;59(7):1234-1238.

© 2024 Schmit N et al. *JAMA Network Open*.
