# Supplementary material for: Mosquito Bed Net Use and Burkitt Lymphoma Incidence in Sub-Saharan Africa: A Systematic Review and Meta-Analysis
Source: JAMA Netw Open. 2024 Apr 18;7(4):e247351. doi: 10.1001/jamanetworkopen.2024.7351 (PMC12527478; doi:10.1001/jamanetworkopen.2024.7351)
Supplement: Supplement 2. — Data Sharing Statement [file jamanetwopen-e247351-s002.pdf]

Data Sharing Statement

Schmit. Mosquito Bed Net Use and Burkitt Lymphoma Incidence in Sub-Saharan Africa. *JAMA Netw Open*. Published April 18, 2024. doi:10.1001/jamanetworkopen.2024.7351

Data
Data available: Yes
Data types: Other (please specify)
Additional Information: All data reported in this study were extracted from published studies. The Burkitt lymphoma dataset is available in the Supplementary Material (eTable 3). All covariate estimates used in the analysis are publicly available from the sources cited in the article.
How to access data: All data reported in this study were extracted from published studies. The Burkitt lymphoma dataset is available in the Supplementary Material (eTable 3). All covariate estimates used in the analysis are publicly available from the sources cited in the article.
When available: With publication

Supporting Documents
Document types: None

Additional Information
Who can access the data: All secondary data used in this study are publicly available.
Types of analyses: All secondary data used in this study are publicly available.
Mechanisms of data availability: All secondary data used in this study are publicly available.
